# A platform for high-throughput screening of DNA-encoded catalyst libraries in organic solvents†
†Electronic supplementary information (ESI) available: Supplemental figures, supporting data, detailed experimental methods, and molecular characterisations. See DOI: 10.1039/c7sc02779f



**DOI:** 10.1039/c7sc02779f

**Published:** 2017-08-21

**Authors:** K. Delaney Hook, John T. Chambers, Ryan Hili

**Affiliations:** a Department of Chemistry , University of Georgia , Athens , GA 30602 , USA . Email: rhili@yorku.ca ; http://www.yorku.ca/rhili/; b Department of Chemistry , York University , Toronto , ON M3J 1P3 , Canada

## Abstract

We have developed a novel high-throughput screening platform for the discovery of small-molecules catalysts for bond-forming reactions. The method employs an *in vitro* selection for bond-formation using amphiphilic DNA-encoded small molecules charged with reaction substrate, which enables selections to be conducted in a variety of organic or aqueous solvents. Using the amine-catalysed aldol reaction as a catalytic model and high-throughput DNA sequencing as a selection read-out, we demonstrate the 1200-fold enrichment of a known aldol catalyst from a library of 16.7-million uncompetitive library members.

## Abstract 2

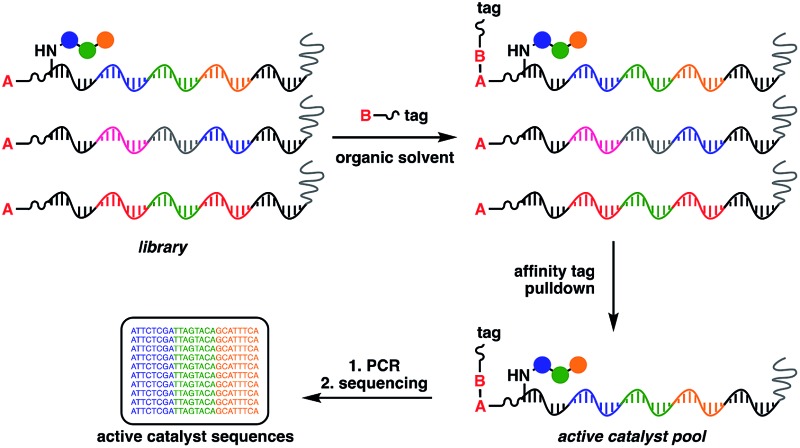
PEGylation of DNA-encoded libraries enables high-throughput screening of small-molecule catalysts in organic solvents.

## Introduction

The *en masse* screening of large combinatorial chemical libraries for catalytic activity provides unique advantages over conventional screening platforms that rely upon methods of discrete synthesis and analysis. Such methods enable the screening of all library members simultaneously for catalytic activity, thus greatly increasing the throughput of catalyst discovery. One notable strategy for high-throughput combinatorial screening of small-molecule catalysts includes the one-bead-one-compound (OBOC) approach,^1^ which relies upon on-bead split-and-pool combinatorial synthesis of chemical libraries and subsequent on-bead or off-bead evaluation. This strategy has resulted in the recent discovery of catalysts capable of effecting chemo- and regioselective modifications on complex targets, including the site-selective epoxidation of polyenes,^2,3^ and reduction of polyunsaturated aldehydes.^4^ One of the major limitations of OBOC and other existing methods for catalyst discovery is the level of throughput. Without advanced infrastructure, multiwell and on-bead screening approaches generally limit the library size to less than 10^4^, with most libraries typically being on the order of 10^3^ or smaller. Approaches that can considerably increase the throughput of catalyst screening, while still enabling the power to multiplex the small molecule libraries for different reaction conditions and transformations, should greatly accelerate the discovery of small-molecule catalysts and structure–activity relationships.

An alternative *en masse* screening method is one that implements a selection pressure to remove catalytically inactive molecules from the library, thus leaving only the catalytically active species to identify. Selections offer significant advantages over traditional screening approaches including (i) a selection evaluates all molecules of a library simultaneously, regardless of library size^5–8^ and (ii) selections are typically easier to execute as they do not require the spatial separation of library members nor sophisticated equipment. *In vitro* selection has been a highly successful and powerful approach for the discovery of catalytic biopolymers from libraries containing greater than 10^14^ members.^9^ Several variants of *in vitro* selection for biopolymer catalysts exist, including phage display,^10^ mRNA display,^11^ ribosomal display,^12^ and DNA display.^13^ The overarching theme amongst these selection methods is that the biopolymer catalyst (phenotype) is spatially associated with its genetic code (genotype), thus PCR amplification and DNA sequencing can reveal the identity of those biocatalysts that have performed the desired catalytic function to survive the selection pressure. The *in vitro* selection of small-molecules from large DNA-encoded libraries^14^ has also been very successful, particularly in the discovery of small molecule drugs.^15–17^ While the application of this technology to medium-throughput screening of aqueous-tolerant reactions using hybridization-dependent microarrays has been successful,^18^ the application of this technology to the discovery of small-molecule catalysts using *in vitro* selection has remained unexplored. The primary reason for this is likely due to the poor solubility of DNA in non-aqueous solvents, where the majority of catalytic reactions operate. Herein, we demonstrate a new high-throughput selection platform for the discovery of small-molecule organic catalysts for intermolecular bond-forming reactions using DNA-encoded libraries in organic solvents.

## Results and discussion

**Fig. 1:**
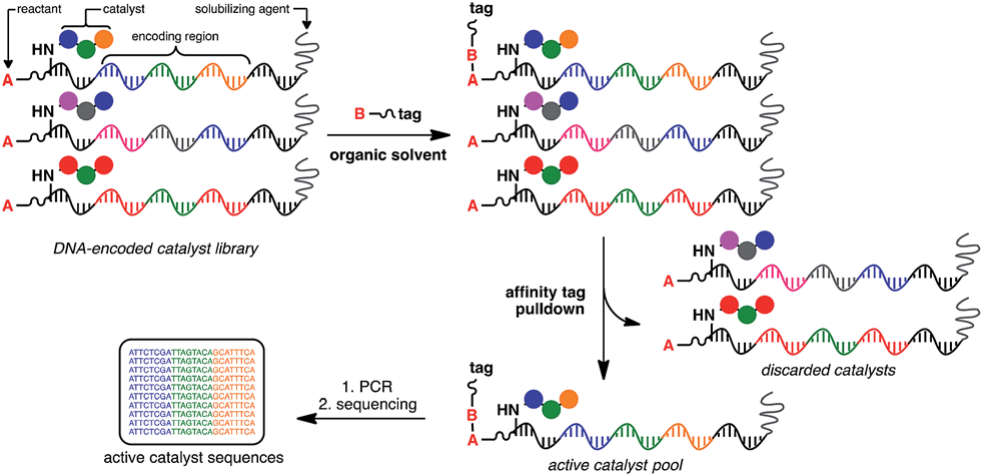
General strategy for the high-throughput screening of catalysts using DNA-encoded libraries in organic solvents.

At the heart of the envisaged platform is a replicable amphiphilic DNA, which can be used to individually barcode members of a large combinatorial chemical library (Fig. 1). The amphiphilic nature of the modified DNA permits solubility in either organic solvents or aqueous media. High-throughput screening for catalytic activity is achieved in organic solvents with only catalytically active members surviving the selection pressure – the ability to catalyse intermolecular bond formation to an affinity tag. The library is then dissolved in aqueous buffer to perform affinity purification, with the surviving members being subjected to PCR amplification and identification by high-throughput DNA sequencing. Since the identity of the small-molecule catalyst can be read directly from the attached DNA barcode, catalytically active library members can be rapidly identified from libraries containing millions of unique members.

The initial step toward realising this high-throughput catalyst-screening platform is the development of a modified DNA that is soluble in both water and commonly used organic solvents. Unmodified DNA is poorly soluble in anhydrous organic solvents, creating heterogeneous mixtures upon aggregation, limiting its use as an encoding element for catalyst selection in organic solvents. Several reports have described methods to increase the solubility of DNA in non-aqueous solvents.^19–29^ While most of these strategies involve the complexation of DNA with surfactants or the generation of nanogels, we were drawn to approaches that conjugated a single polymer to DNA to impart solubility in organic solvents. This would allow for the installation of this solubilising group distal to the site of catalysis, obviating any undesired interactions during catalyst selection. The conjugation of PEG 10 000 to ssDNA has been successful in enabling the solubility of DNA in a variety of organic solvents.^24^ This strategy has only been validated for short oligonucleotide sequences of up to 21 nt in length, which is too short for this selection system; therefore, we sought to determine if this approach could be extended to accommodate longer ssDNAs.

**Fig. 2:**
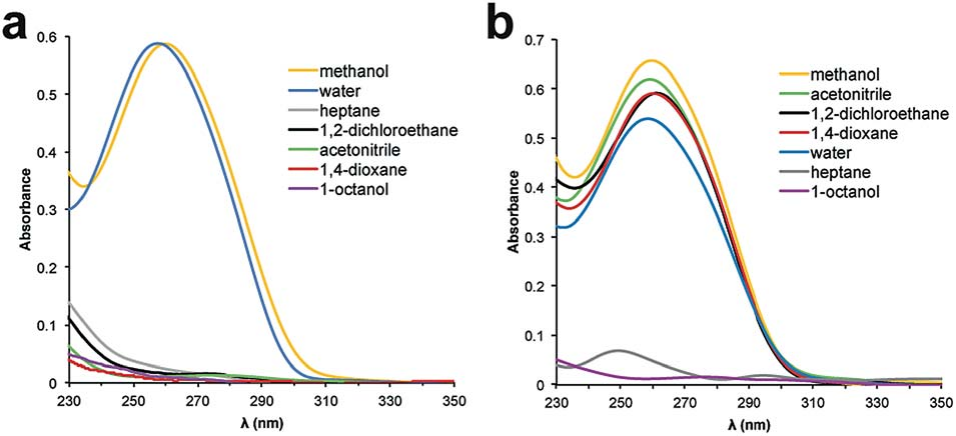
UV-Vis spectra of 48 nt PEGylated ssDNA in various solvents. (a) ssDNA conjugated to PEG 10k. (b) ssDNA conjugated to PEG 40k.

The ssDNA encoding element for the selection platform requires both an encoding region that specifies the catalyst, and two primer regions for amplification. As a model ssDNA length, we chose 48 nucleotides (nt), which accommodates two 18 nt primer sites and a 12 nt catalyst-encoding region; a 12 nt region can encode greater than 16 million unique molecules by established split-and-pool tandem DNA/small-molecule synthesis methods.^30^ To determine the optimal polymer length for our system, we prepared a model 5′-amino modified 48 nt ssDNA sequence, which we conjugated to PEG-*N*-hydroxysuccinimide (PEG-NHS) esters ranging in average mass from PEG 10 000 to PEG 40 000. The influence of a PEG polymer on the solubility of ssDNA in organic solvents was determined by preparing 5 μM solutions of the PEGylated DNA in various solvents and analysing the samples by UV-Vis spectroscopy. Unfortunately, PEG 10 000 was unable to facilitate solubility of the 48 nt ssDNA into any solvents except water and methanol (Fig. 1a). We began to observe partial solubility of PEGylated DNA in 1,2-dichloroethane (DCE) and acetonitrile (MeCN) at PEG weights of 20 000 Da (Fig. S1†); however, an excellent and general solubility profile was observed when using PEG 40 000 (Fig. 2b). Importantly, PEG 40 000 enabled solubility in a range of organic solvents, while maintaining excellent solubility in water. Since nucleobase absorbance can be influenced by solvent effects,^31^ and due to some organic solvents overlapping with the UV absorbance of DNA, we independently quantified solubility using qPCR (Fig. S2†).

**Fig. 3:**
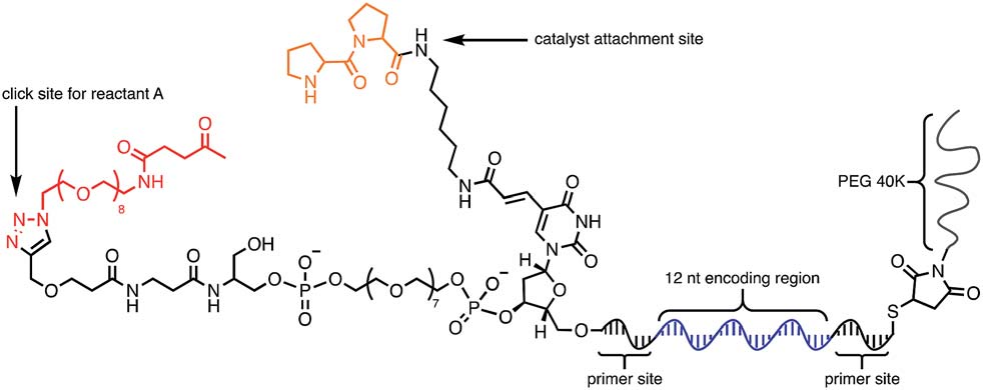
Optimised molecular architecture of the amphiphilic DNA-encoded catalyst with aldol substrate. Ketone aldol reactant is shown attached to DNA.

**Fig. 4:**
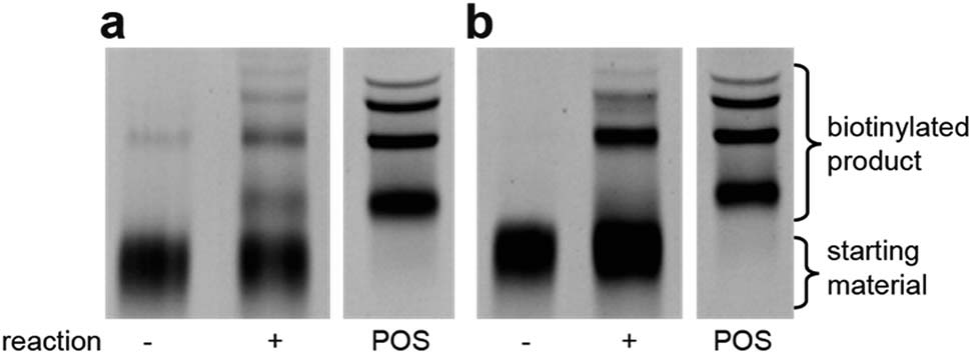
Streptavidin-mediated EMSA comparison of aldol catalysis with either (a) aldehyde reactant on DNA with biotinylated ketone in solution and (b) ketone on DNA with biotinylated aldehyde in solution. Reactions were performed for 5 days in DCE. POS = positive control, biotinylated DNA.

Using the optimised PEG length to permit solubility in organic solvents, we next determined if we could achieve small-molecule catalysis on these amphiphilic DNAs in a variety of organic solvents. Interested in the potential of small peptide catalysts,^32^ and encouraged by the reported success of DNA-templated aldol reactions catalysed by proline-modified ssDNA in aqueous solvents,^33^ we implemented the secondary amine catalysed aldol reaction between a ketone and an aldehyde as our model. We designed a DNA architecture that would accommodate the catalyst site, the reactant site, and a PEGylation site, and could be readily synthesized by solid-phase DNA synthesis. We reasoned that the PEGylation site should be distal to the catalyst and reactant site to minimise interference on catalysis by the PEG chain. We also decided to incorporate a long spacer between the catalyst site and the reactant site to permit sufficient flexibility for the catalyst to comfortably engage the substrate. Thus, a 48 nt PEGylated ssDNA was synthesised to satisfy these specifications (Fig. 3). We chose a 3′-alkynyl group to permit ready conjugation of different aldol reactants by copper-catalysed click reaction.^34^ A flexible PEG spacer was used to separate the aldol reactant and the diproline catalyst. This was followed by a 3′-end primer-binding site, a 12 nt encoding region, and a 5′-end primer-binding site. At the 5′-terminus was installed a thiol, which was used for conjugation to PEG 40 000 maleimide. Using the model with a ketone conjugated to DNA (Fig. 3) and biotinylated benzaldehyde derivative in solution, we sought to conduct the catalytic reaction at concentrations that were likely to be used during a selection. Previous reports of optimised aldol catalysis on DNA templates in aqueous media involved molar concentrations of one of the aldol reactants.^33^ This high concentration was not feasible for selection experiments; for our initial screening of catalyst activity we held the biotinylated aldol reactant at 500 μM with the ssDNA template at 0.5 μM. Characterisation of the catalytic aldol reaction was performed using a streptavidin-based electrophoretic mobility shift assay (EMSA). Reaction success differentiates the product *via* biotin tag which, after incubation with streptavidin, allows visual comparison by a mobility shift between unreacted starting material and successfully catalysed reaction products using native gel electrophoresis. To determine the optimal reaction conditions, a solvent screen was performed for the aldol reaction in the various solvents previously concluded to efficiently solubilise the DNA-encoded catalyst architecture (Table 1). Yields of the catalytic aldol varied greatly depending on the solvent. DCE was found to be the optimal solvent for the process, with solvents such as DMF and DMSO yielding only trace amount of desired product. Since the catalyst-selection system for bond-forming reactions can have either reactant immobilised on DNA, we chose to examine the effect of aldol substrate identity conjugated to the DNA-encoded catalyst. To do this, we prepared both the ketone-conjugated DNA and the aldehyde-conjugated DNA architectures and subjected them to reaction in DCE with the biotinylated aldehyde and biotinylated ketone, respectively. EMSA analysis showed that catalytic bond formation proceeded with both architectures (Fig. 4). These preliminary experiments demonstrated that small-molecule catalysts tagged with a PEGylated DNA could catalyse the aldol reaction in organic solvents, which was a necessary step toward developing a DNA-encoded catalyst selection system in organic solvents.

**Table 1:** Solvent screen for DNA-encoded catalyst activity

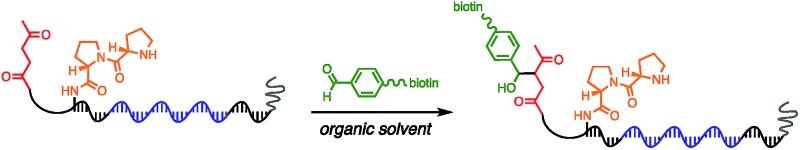		
Entry	Solvent	Yield^*a*^
1	DMSO	<5%
2	DMF	<5%
3	H_2_O	8%
4	1,4-Dioxane	10%
5	MeOH	27%
6	MeCN	27%
7	DCE	43%

^*a*^Ketone–catalyst DNA (0.5 μM), biotinylated benzaldehyde (500 μM), reactions shaken for 5 days at room temperature. Yield was determined by PAGE.

**Fig. 5:**
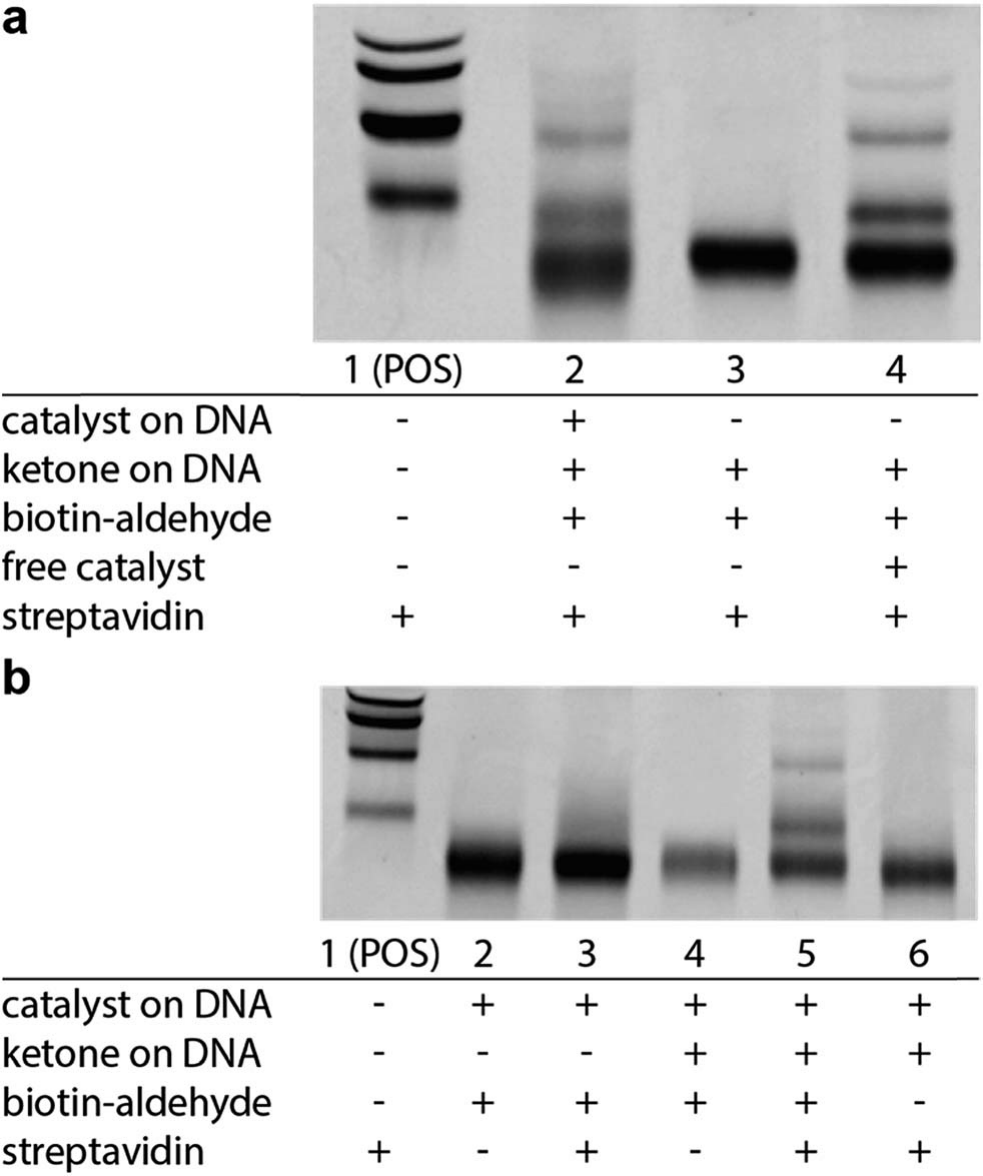
EMSA experiments demonstrating selective catalysis. (a) Aldol reaction depends on the presence of a catalyst on DNA (Lane 2 *vs.* Lane 3). Aldol reaction is rescued by the addition of 1 mM pyrrolidine as a catalyst (Lane 4). (b) EMSA shift requires attached substrate (Lane 3 *vs.* Lane 5) and requires aldol reactant in solution (Lane 5 *vs.* Lane 6). Reaction conditions: DNA (0.5 μM) was dissolved in of DCE. Biotinylated benzaldehyde (500 μM) was added and the reaction were shaken for 5 days at room temperature; pyrrolidine (1 mM) was added when applicable. POS = positive control, biotinylated DNA.

**Fig. 6:**
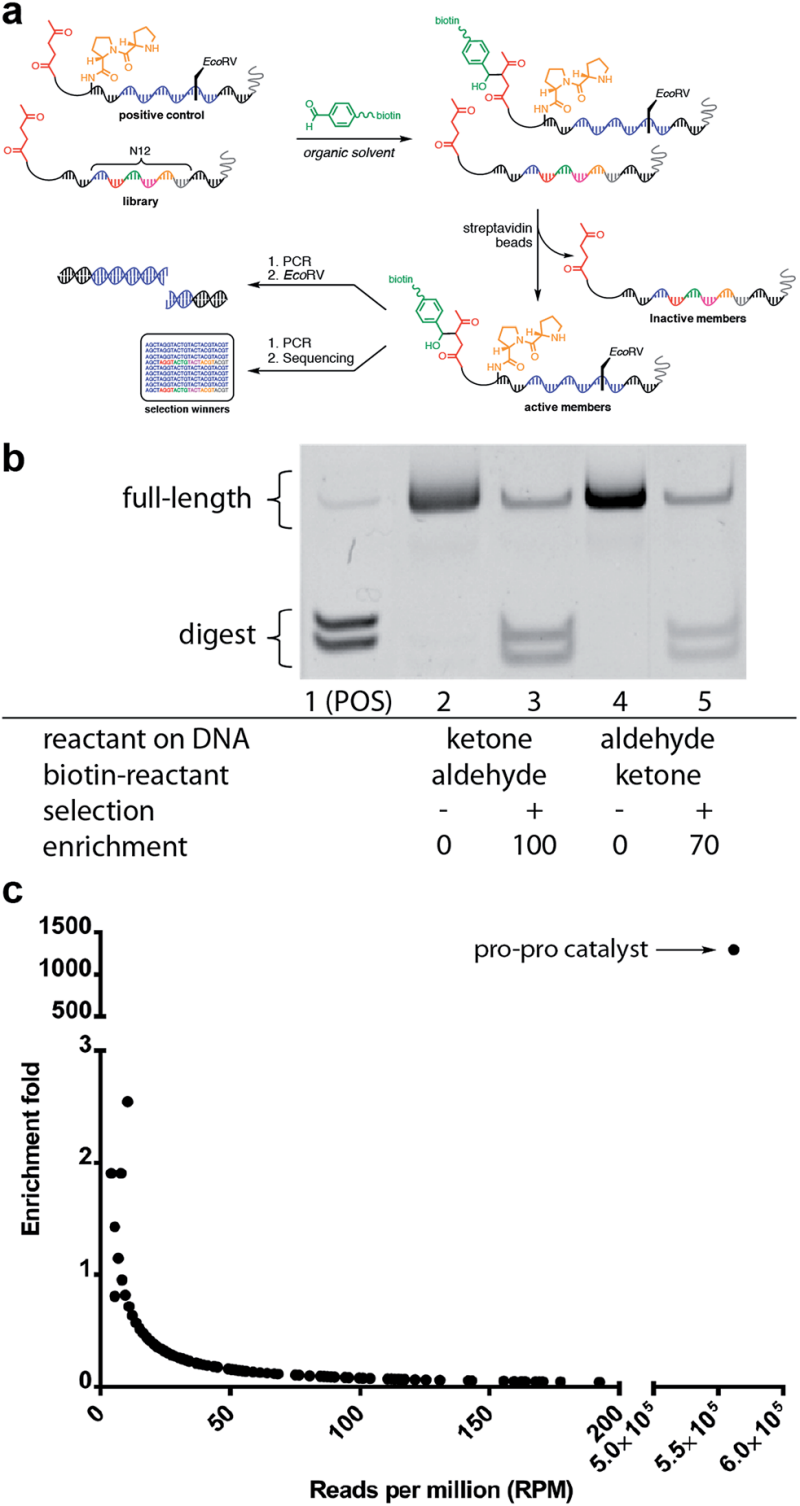
Mock selection for aldol catalysis with DNA-encoded small-molecule libraries. (a) General scheme for the *in vitro* mock selection of a DNA-encoded aldol catalyst. (b) Gel analysis of mock aldol selection resulting from a 500-fold dilution of DNA-encoded aldol catalyst. (c) High-throughput sequencing analysis of mock aldol selection resulting from a 2000-fold dilution of DNA-encoded aldol catalyst.

We next sought to determine if this platform would enable the selective enrichment of active DNA-encoded catalysts from a large library of DNA-encoded molecules. There were several issues that might diminish the enrichment of the known aldol catalyst during the selection, including: (i) DNA bases non-specifically react to form stable covalent adducts with the biotinylated aldol reactant; (ii) DNA catalyses the aldol reaction and results in non-specific biotinylation of DNA; (iii) the catalyst forms stable covalent adducts with the biotinylated aldol reactant; and (iv) inter-strand catalysis results in biotinylation of inactive library members by an active member. To address issues i–iii control experiments were designed to demonstrate that catalysis and bond-formation with biotinylated reactant happens only when the DNA molecule has both the catalyst and the aldol reactant attached (Fig. 5). To address issue iv, and effectively the promise of this method, we designed a selection system with a restriction digest-based readout to assess the enrichment of the catalyst. We implemented a selection pressure whereby survival of a library member required its ability to catalyse the aldol reaction. As a model selection, we sought to enrich the diproline positive control sequence from a large library of uncompetitive members. Each member of the library contained a ketone reactant at the 3′-end. Importantly, the diproline positive control contained an *Eco*RV restriction digest site within its encoding region, which enabled monitoring of its enrichment after the selection round by restriction digest and PAGE analysis (Fig. 6a).

The positive control was diluted 500-fold into a library of DNA sequences that lacked a catalyst. The library mixture was incubated with the biotinylated aldehyde reactant in DCE for three hours followed by binding to streptavidin-coated magnetic beads. After extensive washing of the beads, on-bead PCR was performed to amplify the selection survivors. Enrichment analysis was determined by restriction enzyme digestion of the PCR product, followed by non-denaturing PAGE analysis. After one round of selection for catalytic activity, the positive control was enriched 100-fold (Fig. 6b). Importantly, when the aldol reactants were exchanged, such that the aldehyde was on the DNA template, and the ketone was biotinylated in solution, similar enrichment values (70-fold) were observed (Fig. 6b).

Satisfied with the outcome of the preliminary mock selections, high-throughput DNA sequencing was performed to quantitatively determine the fold enrichment of the aldol reaction selection under more dilute selection conditions. Compared to the EMSA characterisation, which only allows characterisation of one specific sequence, DNA sequencing permits characterisation of all the sequences in a library allowing for a more in-depth analysis of the selection outcome. Aldol selection was performed as described above with the positive control diluted 2000-fold into a library of 16.7 million (N_12_) library members; however, instead of restriction digest as a readout, Illumina barcoded adapters were added to the template sequences by PCR amplification and Illumina Mi-Seq paired-end sequencing was performed (Fig. 6). Post-sequencing analysis (see ESI†) involved merging of paired-end reads and trimming off sequencing adapters to yield readouts of the survivors of the aldol selection. By comparing the sequence frequencies of the starting library with those of the post-selection library, enrichment levels could be readily calculated for each sequence (Fig. 6c). Sequencing analysis revealed that the positive control diproline catalyst was strongly enriched by 1200-fold. This level of enrichment suggests that this method could support the *de novo* discovery of small-molecule catalysts for bond-forming reactions.

## Conclusions

In summary, we have developed a catalyst selection system based upon the use of DNA-encoded libraries in organic solvents. Survival of the selection requires DNA-encoded catalysts to engage in catalytic bond formation between an *in-cis* reactant and an *in-trans* biotinylated reactant. Affinity pull-down and readout by high-throughput DNA sequencing enables the rapid identification of active catalysts. Using the amine-catalysed aldol reaction as a model, we demonstrated that this approach can be implemented in various organic and aqueous solvents and can enrich a known aldol catalyst by 1200-fold. This platform has the potential to greatly accelerate the discovery of catalysts by increasing the throughput of catalyst screening efforts and expanding the chemical space explored during conventional catalyst screenings.

## Conflicts of interest

There are no conflicts to declare.
