# Supplementary material for: A platform for high-throughput screening of DNA-encoded catalyst libraries in organic solvents
Source: Chem Sci. 2017 Aug 21;8(10):7072–6. doi: 10.1039/c7sc02779f (PMC5637469; doi:10.1039/c7sc02779f)
Supplement: Supplementary file 1 [file SC-008-C7SC02779F-s001.pdf]

Electronic Supplementary Material (ESI) for Chemical Science.
This journal is © The Royal Society of Chemistry 2017

Supporting Information for:

A Platform for High-Throughput Screening of DNA-Encoded Catalyst Libraries in Organic Solvents

K. Delaney Hook, John T. Chambers, and Ryan Hili*

Department of Chemistry, University of Georgia, Athens, Georgia, 30602

Table of Contents

Supporting Data

Figure S1. UV-vis spectra of 20,000 MW PEGylated DNA in organic solvents S2

Figure S2. qPCR solubility data of 40,000 MW PEGylated DNA in organic solvents S2

Figure S3. Aldol reaction selection outcome with control S3

Table S1. DNA sequencing results (top 100 selection survivors) S11

Supporting Methods

General Method S3

Oligonucleotide Synthesis S4

DNA Sequences S4

Modified Oligonucleotide Synthesis S5

Small Molecule Synthesis S7

PEG-DNA Solubility S9

Catalyst Selection Procedure S10

DNA Sequencing S11

Related Spectra S15

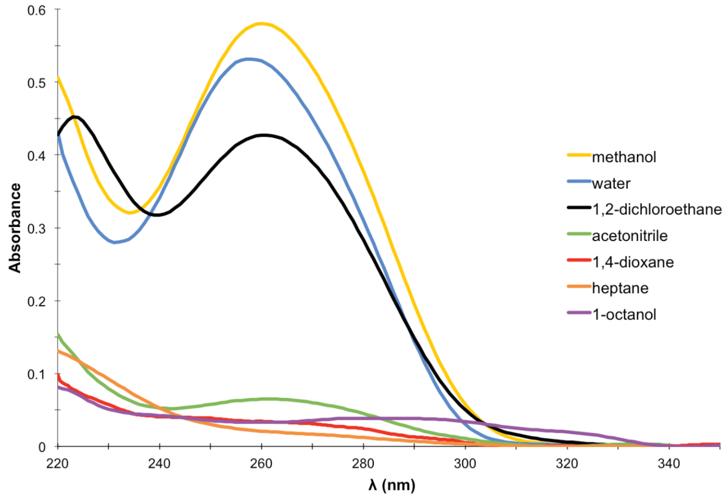

Figure S1. PEG-DNA solubility. UV-vis spectra of 20,000 MW PEGylated DNA dissolved in various organic solvents. Dissolved at 5 μM and diluted to 1 μM for experiment.

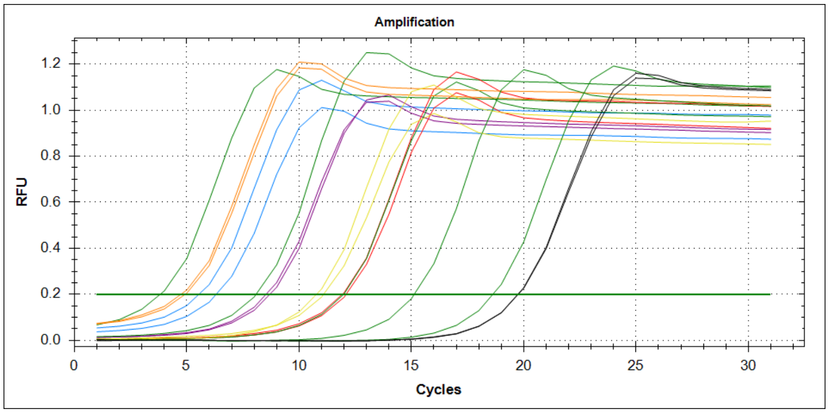

Solvent	Concentration [a]
H_2O	0.90 ± 0.07 μM
DCE	1.0 ± 0.03 μM
DMSO	0.54 ± 0.04 μM
DMF	0.02 ± 0.003 μM
toluene	0.02 ± 0.002 μM
heptane	- [b]

Figure S2. qPCR solubility analysis of 40,000 MW PEGylated DNA in various solvents (DMSO, DMF, toluene, 1,2-dicholorethane, water, and heptane). Samples dissolved at 1.0 μM with experiments performed in triplicate. [a] concentration determined by fit to a standardized concentration amplification curve. [b] amplification begins after no template control (NTC) amplification.

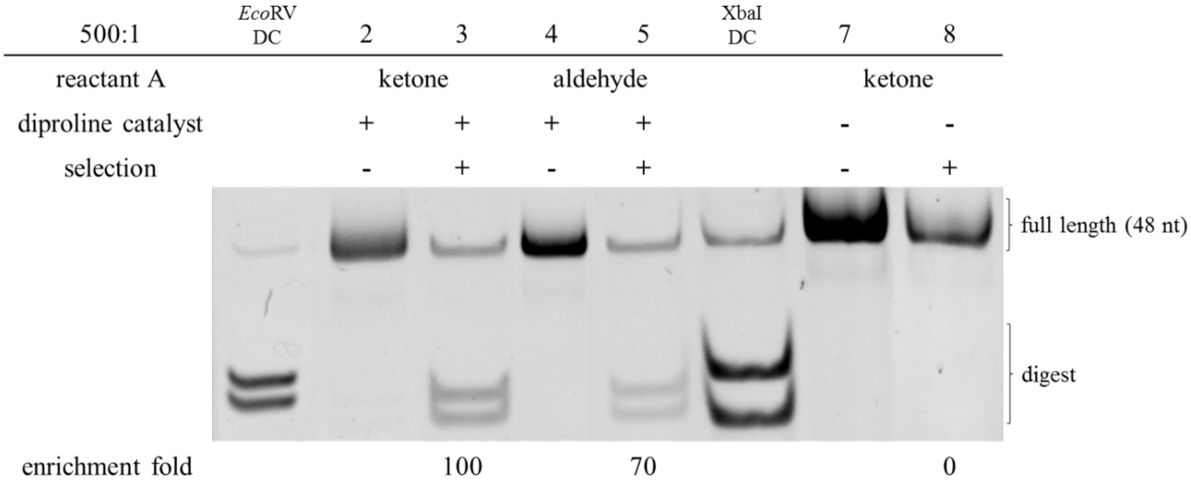

Figure S3. Aldol reaction selection outcome. Lanes 1-5: catalyst DNA (Ta_Eco_RV encoded). Lane 1: Eco*RV* digest control. Lane 2 Starting library with 3'-ketone (500:1 non-competitive/no-catalyst vs diproline catalyst encoded DNA). Lane 3: Selection outcome with ketone on DNA selection. Lane 4: Starting library with 3'-aldehyde (500:1 non-competitive/no-catalyst vs diproline catalyst encoded DNA). Lane 5. Selection outcome with aldehyde on DNA selection. Lanes 6-8: no catalyst DNA (Ta_NC encoded). Lane 7: 500:1 starting library with 3'-ketone N12 library vs 3'-ketone Ta_NC encoded DNA (no catalyst). Lane 8: Negative control (lane 7) selection outcome. DC = restriction enzyme digest control. Negative control experiments performed with different encoding sequence. Samples were run on 15% non-denaturing PAGE.

General Method

Modified oligonucleotides were synthesized on controlled pore glass (CPG) solid support with a 12-port MerMade automated DNA synthesizer (Bioautomation Corporation). dA/C/G/T phosphoramidites were purchased from Bioautomation Corporation. Modified phosphoramidites were purchased from Glen Research. PEG reagents were purchased from JenKem Technology. Oligonucleotides were purified by RP-HPLC on Hydrosphere C18 column (YMC). All solvents used for solubility measurements were of HPLC grade and purchased from Sigma-Aldrich and dried by activated 3 Ǻ molecular sieves. Organic solvent solubility absorbance spectra were measured on a Thermo-Scientific Evolution 260 Bio UV-Visible Spectrophotometer. Routine concentrations of oligonucleotides analyzed absorbance on NanoDrop

spectrophotometer (Thermo Scientific). All NMR spectra were recorded in $CDCl_3$ using 400 MHz instrument. RNAse-free water was used for all experiments.

Oligonucleotide Synthesis. Phosphoramidites were coupled on solid support 3'alkyne-modifier serinol CPG resin (Glen Research). Modified phosphoramidites used include Thiol-modifier C_6 S-S, spacer phosphoramidite 18, and Amino Modifier C6 dT (Glen Research). Normal phosphoramidites purchased from Bioautomation Corporation. dA- + dC- + dG- + dT- CE phosphoramidites mix was used for 'N' amidites (Glen Research). All modifiers were used as recommended by manufacturer.

Syntheses performed on 1 μmol scale on Mermade 12 DNA Synthesizer (Bioautomation Corporation) with final DMT on. Dried support was transferred to Eppendorf tubes and incubated in fresh ammonium hydroxide (400 μL each) at room temperature for 36 hours with rotation for solid support cleavage and nucleobase deprotection. The solution was filtered from resin, concentrated on speed vacuum, and purified by RP-HPLC. DMT deprotection was performed in 80% acetic acid (400 μL) for 1 hr at room temp, frozen, lyophilized, and purified by RP-HPLC. Product fractions were collected, and lyophilized overnight to yield functionalized oligonucleotides.

DNA Sequences

	(5'→3')
Ta_N12	/5ThioMC6-DS/CGTACGGTCGACGCTAGC *NNNNNNNNNNNN* CACGTGGAGCTCGGATCC/iAmMC6T//iSp18//3AlkSer/
Ta_EcoRV	/5ThioMC6-DS/CGTACGGTCGACGCTAGC *TGGATATCACTG* CACGTGGAGCTCGGATCC/iAmMC6T//iSp18//3AlkSer/
Ta_NC	/5ThioMC6-DS/CGTACGGTCGACGCTAGC *ATGTCCAGTTAG* CACGTGGAGCTCGGATCC/iSp18//3AlkSer/
PFa (forward primer)	CGTACGGTCGACGCTAGC
PRa (reverse primer)	GGATCCGAGCTCCACGTG

Modified Phosphoramidite Codes

/3AlkSer/	3' Alkyne Modifier Serinol CPG
/iSp18/	Int Spacer 18
/iAmMC6T/	Int Amino Modifier C6 dT
/5ThioMC6-DS/	5' Thiol Modifier C6 S-S (Disulfide)

Modified Oligonucleotide Synthesis

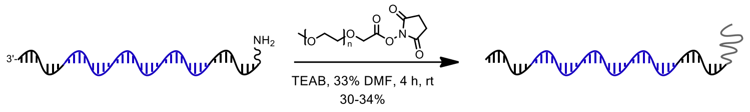

PEGylation of 5'-amino modified oligonucleotides (solubility experiments). To a 5'-amino modified oligo (40 µL, 500 µM, 20 nmol) was added TEAB Buffer, pH 8.7 (40 µL, 177 mM) and 10,000, 20,000, 30,000, or 40,000 MW PEG-NHS ester (100 eq.) dissolved in 40 µL of DMF. The reaction was incubated at room temperature overnight, and purified by RP-HPLC [retention time (rt) = 21 min].

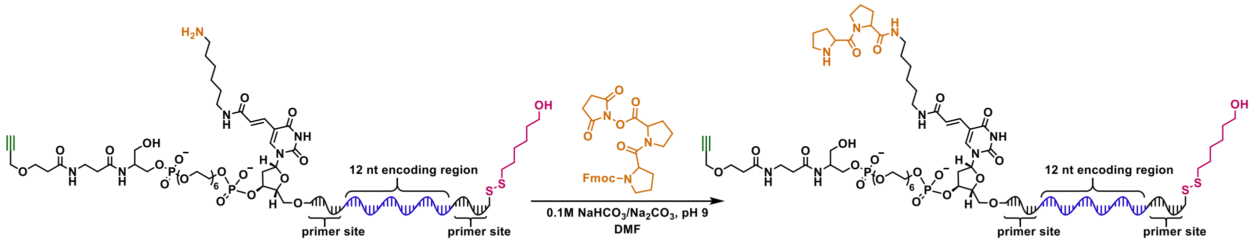

Proline-proline coupling on amino-modified oligonucleotide. Fmoc-pro-proline NHS ester (10 mg/ml, ~20 mM in DMF) was prepared fresh and added to amino-modified oligo (20 nmol, 500 µM) solution in 10 X conjugation buffer ($NaHCO_3/Na_2CO_3$, pH 9, 1 M). Mixture was vortexed and incubated at room temperature for 2 hr. Crude mixture was evaporated to dryness, purified by size exclusion column and evaporated to dryness again. Product was Fmoc deprotected in 20 % piperidine/DMF for 10 min at room temperature and evaporated to dryness, deprotection repeated. Product was purified by HPLC on Hydrosphere C18 column to yield the diprolinamide bound oligonucleotide (6.26 nmol, 31.3 % yield, rt = 17.8 min).

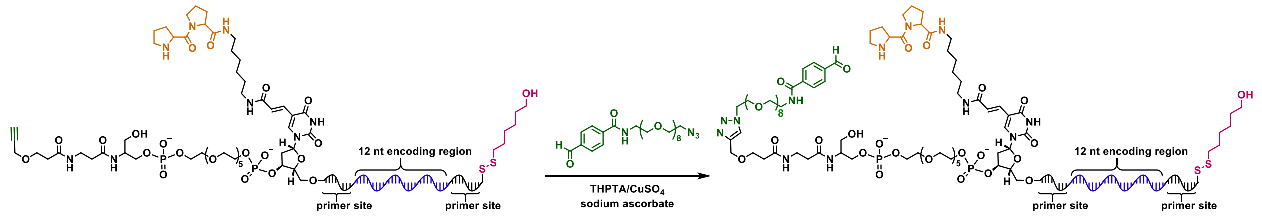

Azido-ketone (4) or benzaldehyde (3) reactant click conjugation. Alkyne oligo (25 nmol, 500 µM) was combined with 10 mM azido-PEG-ketone (1000 nmol, 40 eq.), THPTA/$CuSO_4$ (pre-chelated, 100 mM/50 mM, 625 nmol, 25 eq.), and sodium ascorbate (100 mM, 1250 nmol, 50 eq.) and rotated for 1hr at room temperature. Reaction mixture was filtered through gel column to remove salts and copper sulfate and purified by RP-HPLC on hydrosphere column (rt = 19.9 min) to produce 12.78 nmol (51.1% yield).

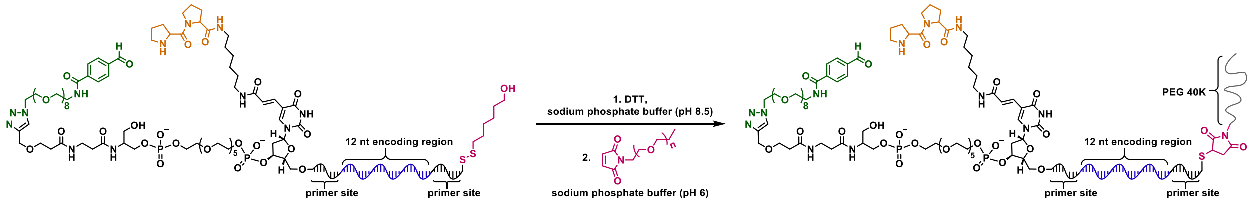

PEGylation of 5'-disulfide oligonucleotides. Disulfide oligo (11 nmol) was incubated with 100 mM DTT (125 µL) in sodium phosphate buffer (50 mM, pH 8.5) for 1 hr. Residual salts and DTT were removed by Princeton separation column preloaded with phosphate buffer (50 mM, pH 6.0). Activated sulfhydryl oligo was immediately combined with 40K PEG maleimide (130 eq., 20 mM in pH 6 phosphate buffer), vortexed, and incubated at 4 °C overnight. Reaction mixture was purified by RP-HPLC on hydrosphere column (rt = 21 min) to produce 3.59 nmol (32.6 % yield).

Mass Spectrometry of Modified Oligonucleotides

Oligonucleotide		Calculated Mass	LC-ESI-MS
YAm48DS	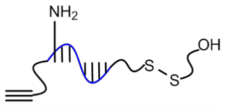	16,181.96	16,185.6 (+3.64)
YPP48DS	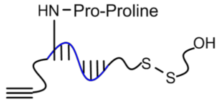	16,377.07	16,376.0 (-1.07)
KPP48DS	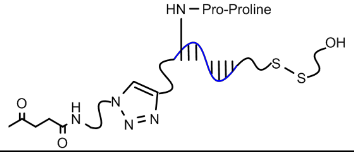	16,931.69	16,914.0 (+0.31)
APP48DS	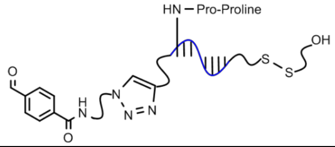	16,947.71	16,948.1 (+0.93)

Y = alkyne Am = amino DS = disulfide PP = proline-proline K = ketone A = aldehyde

Small Molecule Synthesis

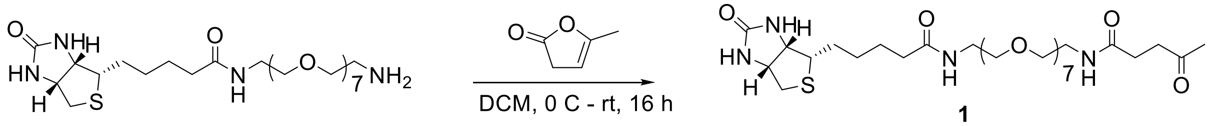

1

Biotinylated ketone (1). α-Angelica lactone (8.92 µL, 0.1 mmol) dissolved in 200 µL CH_2Cl_2 was cooled to 0 °C. Upon addition of Biotin-PEG-amine (59.5 mg, 0.1 mmol) the reaction was warmed to room temperature and stirred overnight. Crude reaction mixture was directly loaded onto silica gel column and eluted with 5-10% MeOH in CH_2Cl_2. Product fractions were collected and concentrated *in vacuo* to yield 20.8 mg of biotin-PEG-ketone as a yellow oil (30 % yield). ^{1}H-NMR (400 MHz, $CDCl_3$): δ (ppm) 4.61 (m, 1H), 4.42 (m, 1H), 3.56-3.64 (m, 28H), 3.35-3.45 (m, 3H), 3.17-3.20 (m, 2H) 2.91-2.95 (m, 1H), 2.80-2.84 (m, 3H), 2.47 (t, 2H,), 2.29-2.32 (m, 2H), 2.18 (s, 3H), 1.6-1.8 (m, 4H), 1.45-1.48 (m, 2H). ESI-MS: $C_{31}H_{56}N_4O_{11}SCl^-$ $[M-Cl]^-$ calculated 727.34, found 727.3.

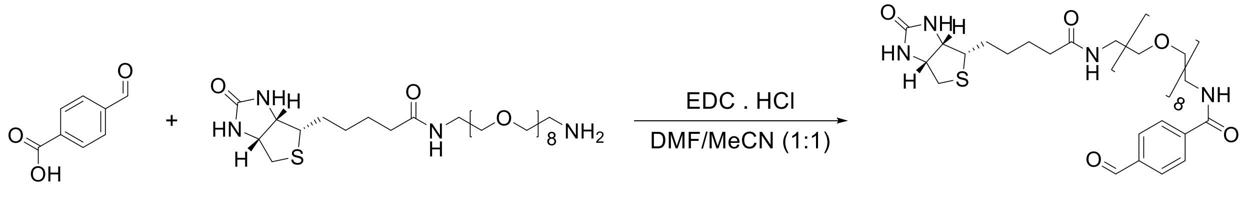

2

Biotinylated aldehyde (2). 4-formyl benzoic acid (26.4 mg, 0.18 mmol, 2.1 eq.) and EDC-HCl (35.5 mg, 0.19 mmol, 2.2 eq.) were weighed into 10 mL round bottom flask and purged with nitrogen gas. To the biotin-PEG-amine (50 mg, 0.08 mmol) was added 500 μL of DMF/MeCN (1:1) and stirred until dissolved. The biotin-PEG-amine solution was slowly added to the reaction vial followed by 1.18 mL of DMF/MeCN for a total volume of 1.68mL. The reaction was stirred at room temperature for 3 hr (reaction completeness monitored by TLC). The solvent was removed *in vacuo*; Crude product was dissolved in DCM and loaded onto silica gel column and eluted with 0-10% MeOH in CH_2Cl_2. Product fractions were collected and concentrated *in vacuo* to yield 20.90 mg (0.0288 mmol, 34% yield) of biotin-PEG-aldehyde as a yellow oil. Rf = 0.36 in 10% MeOH/DCM. ^{1}H-NMR (400 MHz, $CDCl_3$): δ (ppm) 10.06 (s, 1H), 7.92-7.99 (m, 4H), 7.15 (br. s, 1H), 3.36-3.67 (m, 36 H). ESI-MS: $C_{34}H_{54}N_4O_{11}$ $[M + Na]^+$ calculated 749.34, found 749.4.

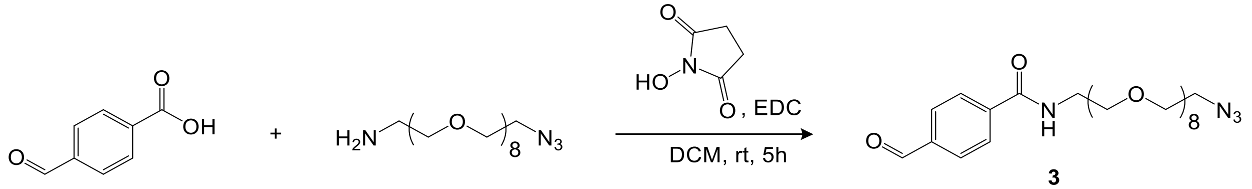

3

Azido-PEG-aldehyde (3). Azido-PEG-amine (110 mg, 0.25 mmol, 0.5M) was added to 4-formyl benzoic acid (75 mg, 0.5 mmol, 2.0 eq.), N-hydroxysuccinimide (57.5 mg, 0.5 mmol, 2.0 eq.), and EDC (100.7 mg, 0.525 mmol, 2.1 eq.) in 0.5 mL DCM. Reaction mixture was stirred at room temperature for 5 hr, diluted with 5 mL of DCM and 5 mL of 1M HCl. Organic layer was separated, dried with magnesium sulfate, and concentrated on rotary evaporator. Mixture was purified by silica gel chromatography (DCM to 10% MeOH in DCM) to yield a yellow oil (25.64 mg, 19% yield). ^{1}H-NMR (400 MHz, $CDCl_3$): δ (ppm) 10.06 (s, 1H), 7.92-7.99 (m, 4H), 7.15 (br. s, 1H), 3.36-3.67 (m, 36 H). ESI-MS: $C_{26}H_{42}N_4O_{10}$ $[M+H]^+$ calculated 571.29, found 573.2.

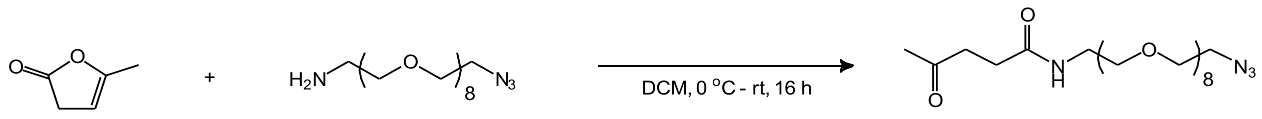

4

Azido-PEG-ketone (4). α-Angelica lactone (22.5 μL, 0.25 mmol) dissolved in 0.5 mL of CH_2Cl_2 was cooled to 0 °C. Azido-PEG-amine (0.25 mmol) was added to the solution and allowed to warm to room temperature. Reaction was stirred under nitrogen overnight. Crude reaction mixture directly loaded into silica gel column and purified using a solvent gradient of 0-10% MeOH in EtOAc to afford the product (60 mg, 44.7% yield). ^{1}H-NMR (400 MHz, $CDCl_3$): δ (ppm) 6.26 (br. s, 1H), 3.37-3.68 (m, 36 H), 2.78 (t, 2H), 2.42-2.45 (t, 2H), 2.17 (s, 3H). ESI-MS: $C_{23}H_{44}N_4O_{10}$ $[M+H]^+$ calculated 537.31, found 537.2.

PEG-DNA Solubility

Measurement of PEGylated DNA by UV-Vis spectrophotometry. 10,000-40,000 MW PEGylated-DNA (500 pmol) was lyophilized in Eppendorf tubes. 100 μL of H_2O, methanol, acetonitrile, 1,4-dioxane, 1,2-dicholorethane, heptane, or 1-octanol was added to dry PEG-DNA and vortexed overnight to ensure complete dissolution. Samples were mildly centrifuged. 5 μL of solution was diluted with 95 μL of solvent [1 μM] and absorbance was measured at 260 nm.

Measurement of PEGylated DNA solubility in UV-active solvents by qPCR. A 10-fold dilution standard curve of aqueous phase oligo was prepared and tested in tandem with duplicate samples of the PEG-oligo in water, DMSO, DMF, 1,2-dichloroethane, toluene, and heptane. The initial sample of DNA contained a 5 μM solution (500 pmol in 100 μL solvent). Once dissolved and gently centrifuged to compact any solid suspension, 5.0 μL was removed, evaporated to in speed vacuum, and dissolved in 10 μL of water [0.1 μM]. The sample was serially diluted by adding 10 μL sample to 90 μL of water to a concentration of 1 nM. 1.0 μL of this solution was combined with SsoAdvanced polymerase (Bio-Rad). Thermocycling and fluorescence measurements were performed on MiniOpticon Real-Time PCR System (Bio-Rad).

Catalyst Selection Procedure

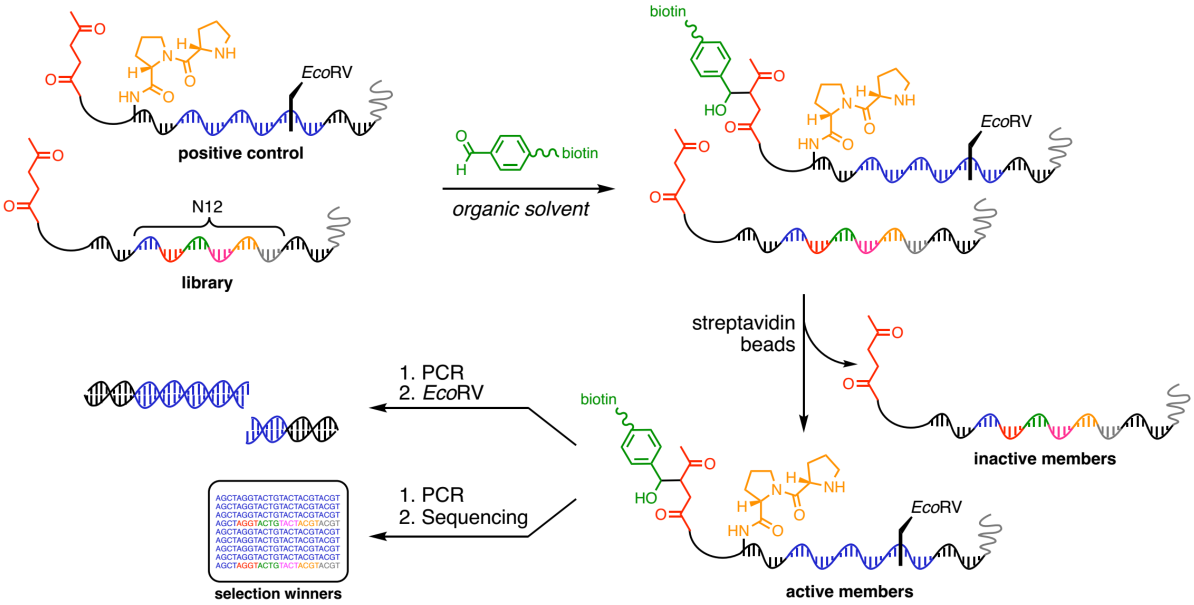

Aldol Reaction. Ketone-ProProline-PEG or Aldehyde-ProProline-PEG 48mers were concentrated in speed vacuum from aqueous solutions (20 pmol). Dried oligos were dissolved in 30 μL of various reaction solvents and shaken overnight to ensure complete dissolution. Biotinylated-aldehyde (**3**) or biotinylated-ketone (**1**) solutions were prepared in each solvent [5 mM] and 4 μL was added to the appropriate reaction vial. Negative control vial contained no biotinylated reactant. Reaction mixtures were vortexed for 90 seconds, spun down, and incubated at 25 ° C for 5 days. Crude reaction samples were evaporated to dryness in speed vacuum, dissolved in 30 μL of water, and excess biotinylated reactant was removed by Princeton Centri-Sep gel filtration column. Products were evaporated to dryness, dissolved in streptavidin buffer solution (2.2 μL, 1 mg/mL), diluted with water to 5 μL, and incubated at room temperature for 30 min. 6x loading dye (1 μL) was added, samples were loaded onto 5% TBE non-denaturing gel, run a 150 V for 90 min, stained with EtBr and imaged.

*Aldol reactions for selection experiments were performed with 100:1, 500:1, and 2,000:1 mixtures of N12-PEG oligos and catalyst-PEG oligos, respectively. Reactions performed as explained above for 3 hours at room temperature.

Streptavidin bead affinity pull-down. Hydrophilic streptavidin-coated magnetic beads (2 µL, Invitrogen) were prewashed and transferred to an Eppendorf tube, buffer removed with beads magnetized, followed by three washes with 500 µL of Wash Buffer (5 mM Tris HCl pH 8, 0.5 mM EDTA, 1M NaCl, 0.05% tween). The buffer was removed and 4 µL (twice the volume of initial beads) of 2X Binding Buffer (10 mM Tris HCl pH 8, 1 mM EDTA, 2M NaCl) was added. Oligo sample (10 pmol, 4 µL in H_2O) added to bead solution and incubated for 30 min with rotation at room temperature. Non-biotinylated oligos were removed with eight washes with Wash Buffer (500 µL) and two washes with Binding Buffer (500 µL). For each wash, the buffer was kept cold and tubes were rotated at room temperature for 5 minutes.

PCR amplification – on bead. Selection product bound magnetic streptavidin beads were serially diluted 5000-fold in DI water. 1 µL of diluted beads was transferred to self-seal cap PCR tubes and combined with forward and reverse primers, PFa and PRa (20 pmol each, 1.0 µM), 2X Hot Start Taq (10 µL), and 5 µL water. The PCR reactions were performed in a thermocycler with the following program: 1. 3 min, 95°C. 2. 30 sec, 95°C. 3. 30 sec, 58 °C. 4. 1 min, 72°C**.** Steps 2-4 were repeated for 20 cycles of PCR. Products were immediately put on ice and purified with Qiagen Minelute PCR purification kit following recommended manufacturer's procedure.

Restriction enzyme digestion. Purified PCR product DNA (0.1 µg) was combined with 10 X Cutsmart Buffer (NEB) (5 µL), restriction enzyme EcoRV (1.0 µL, 20,000 U/mL) (NEB) and diluted to 50 µL with water. Digestion reactions were incubated for 2 hr at 37 $^{\circ}$ C.

DNA Sequencing

Illumina Primers

iTurS_i7_D707C CAA GCA GAA GAC GGC ATA CGA GAT CTG AAG CT GTG ACT GGA GTT CAG ACG TGT GCT CTT CCG ATC TGG ATC CGA GCT CCA CGT G

iTurS_i7_D708C CAA GCA GAA GAC GGC ATA CGA GAT TAA TGC GC GTG ACT GGA GTT CAG ACG TGT GCT CTT CCG ATCT GG ATC CGA GCT CCA CGT G

PRIMEC_D AAT GAT ACG GCG ACC ACC GAG ATC T ACA CTC TTT CCC TAC ACG ACG CTC TTC CGA TCT CGT ACG GTC GAC GCT AGC

Post-Sequencing Analysis

Pre-processing. Raw Illumina paired-end read .fastq files were merged using the PEAR software[1]. The sequences were trimmed to 48nt by removing the sequencing primer codes using a Python script.

Post-processing. All post processing was performed using the FASTAptamer software[2]. The sequences we clustered using a Levenshtein edit distance of 3. Enrichment was determined by comparing normalized reads per million (RPM) values the starting library sequences and the post-selection sequences.

Sequencing Results

Table S1. Top 100 reads of selection readout compared to reads in starting library. Sequence reads normalized to reads per million (RPM).

Rank	Sequence	Initial (RPM)	Selection (RPM)
1	CGTACGGTCGACGCTAGCTGGATATCACTGCACGTGGAGCTCGGATCC	569.58	590795.45
2	CGTACGGTCGACGCTAGCGAAGTTGGTTTACACGTGGAGCTCGGATCC	2.60	38.18
3	CGTACGGTCGACGCTAGCCTAGCTCATTCTCACGTGGAGCTCGGATCC	2.60	79.11
4	CGTACGGTCGACGCTAGCGCTATATCTTATCACGTGGAGCTCGGATCC	2.60	81.83
5	CGTACGGTCGACGCTAGCTACTTCTGTTTACACGTGGAGCTCGGATCC	2.60	80.46
6	CGTACGGTCGACGCTAGCATCATTTTTCACCACGTGGAGCTCGGATCC	2.60	105.02
7	CGTACGGTCGACGCTAGCTTGACAAGCCTTCACGTGGAGCTCGGATCC	2.60	45.00
8	CGTACGGTCGACGCTAGCGGGCTATCGTGTCACGTGGAGCTCGGATCC	2.60	60.01
9	CGTACGGTCGACGCTAGCTGCTGTGTCCGCCACGTGGAGCTCGGATCC	2.60	27.29
10	CGTACGGTCGACGCTAGCATGACCCCCGGCCACGTGGAGCTCGGATCC	2.60	24.55
11	CGTACGGTCGACGCTAGCACAATTTCATCTCACGTGGAGCTCGGATCC	2.60	87.29
12	CGTACGGTCGACGCTAGCACCCAATAACCACACGTGGAGCTCGGATCC	2.60	20.46
13	CGTACGGTCGACGCTAGCCATTTACCTATCCACGTGGAGCTCGGATCC	2.60	50.46
14	CGTACGGTCGACGCTAGCTTTGAGTATAGCCACGTGGAGCTCGGATCC	2.60	32.73
15	CGTACGGTCGACGCTAGCCTAACTACTCCACACGTGGAGCTCGGATCC	2.60	36.82
16	CGTACGGTCGACGCTAGCTCAATAAAGGGGCACGTGGAGCTCGGATCC	2.60	34.10
17	CGTACGGTCGACGCTAGCTAGTCCGTCATGCACGTGGAGCTCGGATCC	2.60	46.37
18	CGTACGGTCGACGCTAGCCGTTATAGTAAACACGTGGAGCTCGGATCC	2.60	51.83
19	CGTACGGTCGACGCTAGCCCGTCTCCTGGACACGTGGAGCTCGGATCC	2.60	34.10
20	CGTACGGTCGACGCTAGCGCTGCTTTATTCCACGTGGAGCTCGGATCC	2.60	38.19
21	CGTACGGTCGACGCTAGCCTGGTCAACTATCACGTGGAGCTCGGATCC	2.60	32.73
22	CGTACGGTCGACGCTAGCTTCAGTTCTTTCCACGTGGAGCTCGGATCC	2.60	75.02
23	CGTACGGTCGACGCTAGCAAATTATCAATCCACGTGGAGCTCGGATCC	2.60	72.29
24	CGTACGGTCGACGCTAGCATGGGTGATCTTCACGTGGAGCTCGGATCC	2.60	16.37
25	CGTACGGTCGACGCTAGCTGGCATACTTGTCACGTGGAGCTCGGATCC	2.60	58.65
26	CGTACGGTCGACGCTAGCCTGCTTCTGTCACACGTGGAGCTCGGATCC	2.60	47.73
27	CGTACGGTCGACGCTAGCTGACAACGATAGCACGTGGAGCTCGGATCC	2.60	20.46

28	CGTACGGTCGACGCTAGCAACTTCACTGTTCACGTGGAGCTCGGATCC	2.60	42.28
29	CGTACGGTCGACGCTAGCTCCCTTATCTGGCACGTGGAGCTCGGATCC	2.60	32.73
30	CGTACGGTCGACGCTAGCTTCACTCTCTACCACGTGGAGCTCGGATCC	2.60	61.37
31	CGTACGGTCGACGCTAGCCAATCCGACACACACGTGGAGCTCGGATCC	2.60	50.46
32	CGTACGGTCGACGCTAGCTCGGTGTTGACGCACGTGGAGCTCGGATCC	2.60	65.47
33	CGTACGGTCGACGCTAGCCTGAGATGTCCCCACGTGGAGCTCGGATCC	2.60	21.83
34	CGTACGGTCGACGCTAGCGGTAAATAACATCACGTGGAGCTCGGATCC	2.60	38.19
35	CGTACGGTCGACGCTAGCATGATAGTCTTACACGTGGAGCTCGGATCC	2.60	36.82
36	CGTACGGTCGACGCTAGCACTCATTCCGGTCACGTGGAGCTCGGATCC	2.60	45.00
37	CGTACGGTCGACGCTAGCAGTATGGAGAAGCACGTGGAGCTCGGATCC	2.60	24.55
38	CGTACGGTCGACGCTAGCATTCATCCCCGGCACGTGGAGCTCGGATCC	2.60	24.55
39	CGTACGGTCGACGCTAGCTACTGTGCATTCCACGTGGAGCTCGGATCC	2.60	68.17
40	CGTACGGTCGACGCTAGCAGCTCTCAGCCCCACGTGGAGCTCGGATCC	2.60	38.19
41	CGTACGGTCGACGCTAGCTGTGCCTCAGAACACGTGGAGCTCGGATCC	2.60	40.91
42	CGTACGGTCGACGCTAGCTAGAACTGTTAGCACGTGGAGCTCGGATCC	2.60	28.64
43	CGTACGGTCGACGCTAGCTCCTCCATACGGCACGTGGAGCTCGGATCC	2.60	27.27
44	CGTACGGTCGACGCTAGCACAGTAGACATGCACGTGGAGCTCGGATCC	2.60	25.92
45	CGTACGGTCGACGCTAGCATAATGCTACACCACGTGGAGCTCGGATCC	2.60	51.83
46	CGTACGGTCGACGCTAGCAATCGGTTCTCCCACGTGGAGCTCGGATCC	2.60	25.92
47	CGTACGGTCGACGCTAGCGGTTATGTTGGGCACGTGGAGCTCGGATCC	2.60	19.09
48	CGTACGGTCGACGCTAGCGCCACGCCGCGGCACGTGGAGCTCGGATCC	2.60	23.18
49	CGTACGGTCGACGCTAGCTGCATATACGAACACGTGGAGCTCGGATCC	2.60	66.82
50	CGTACGGTCGACGCTAGCCCGCTGAGCCCTCACGTGGAGCTCGGATCC	2.60	28.64
51	CGTACGGTCGACGCTAGCTTGCACATTCACCACGTGGAGCTCGGATCC	2.60	47.73
52	CGTACGGTCGACGCTAGCTATATTGCTCTCCACGTGGAGCTCGGATCC	2.60	64.09
53	CGTACGGTCGACGCTAGCCCGTAATCGTAGCACGTGGAGCTCGGATCC	2.60	40.91
54	CGTACGGTCGACGCTAGCTGGGTTTATCTCCACGTGGAGCTCGGATCC	2.60	31.37
55	CGTACGGTCGACGCTAGCTGAATGTCTTAACACGTGGAGCTCGGATCC	2.60	42.28
56	CGTACGGTCGACGCTAGCCGGATAAGTTCACACGTGGAGCTCGGATCC	2.60	27.27
57	CGTACGGTCGACGCTAGCGATTCATCGAGGCACGTGGAGCTCGGATCC	2.60	32.73
58	CGTACGGTCGACGCTAGCATGAATAGATCTCACGTGGAGCTCGGATCC	2.60	73.63
59	CGTACGGTCGACGCTAGCTTATGTTATTTACACGTGGAGCTCGGATCC	2.60	47.72
60	CGTACGGTCGACGCTAGCGACTACATTAAGCACGTGGAGCTCGGATCC	2.60	36.82
61	CGTACGGTCGACGCTAGCTCGAATGGGAGACACGTGGAGCTCGGATCC	2.60	28.64
62	CGTACGGTCGACGCTAGCGGTGCTGCAGTGCACGTGGAGCTCGGATCC	2.60	25.92
63	CGTACGGTCGACGCTAGCCGTGGATTTCTGCACGTGGAGCTCGGATCC	2.60	39.54
64	CGTACGGTCGACGCTAGCATGATCTATAGGCACGTGGAGCTCGGATCC	2.60	32.73
65	CGTACGGTCGACGCTAGCCCAAAAGGCAGTCACGTGGAGCTCGGATCC	2.60	27.27
66	CGTACGGTCGACGCTAGCATCAAACCTAGTCACGTGGAGCTCGGATCC	2.60	58.64
67	CGTACGGTCGACGCTAGCCTTGCATCAGCGCACGTGGAGCTCGGATCC	2.60	34.10
68	CGTACGGTCGACGCTAGCTAGGTTGGAAAGCACGTGGAGCTCGGATCC	2.60	19.09
69	CGTACGGTCGACGCTAGCTAACGGTTTGTCCACGTGGAGCTCGGATCC	2.60	42.28
70	CGTACGGTCGACGCTAGCAATTCTAACAAACACGTGGAGCTCGGATCC	2.60	23.18
71	CGTACGGTCGACGCTAGCTTTACATATTCTCACGTGGAGCTCGGATCC	26.00	98.19
72	CGTACGGTCGACGCTAGCTAACCCGTTCTCCACGTGGAGCTCGGATCC	2.60	46.37
73	CGTACGGTCGACGCTAGCTTGGAAGTACCACACGTGGAGCTCGGATCC	2.60	70.91
74	CGTACGGTCGACGCTAGCCTACCAACGTAACACGTGGAGCTCGGATCC	2.60	24.55
75	CGTACGGTCGACGCTAGCTGCATTTCAGGTCACGTGGAGCTCGGATCC	2.60	35.45
76	CGTACGGTCGACGCTAGCTGAGAACAATCCCACGTGGAGCTCGGATCC	2.60	36.82
77	CGTACGGTCGACGCTAGCCAATCTTACAGCCACGTGGAGCTCGGATCC	2.60	45.02
78	CGTACGGTCGACGCTAGCTTTCTCCGTTTTCACGTGGAGCTCGGATCC	2.60	68.19
79	CGTACGGTCGACGCTAGCGCGGCTTCTGGTCACGTGGAGCTCGGATCC	2.60	19.09
80	CGTACGGTCGACGCTAGCACTGGAACTTATCACGTGGAGCTCGGATCC	2.60	19.09
81	CGTACGGTCGACGCTAGCGGCTAGAGGGGTCACGTGGAGCTCGGATCC	2.60	10.91
82	CGTACGGTCGACGCTAGCACGTTGCGATTTCACGTGGAGCTCGGATCC	2.60	27.27
83	CGTACGGTCGACGCTAGCACGAGCACCTCCCACGTGGAGCTCGGATCC	2.60	39.54
84	CGTACGGTCGACGCTAGCCGCCATCAACACCACGTGGAGCTCGGATCC	2.60	38.19
85	CGTACGGTCGACGCTAGCAGTCTGAATTGGCACGTGGAGCTCGGATCC	2.60	31.36
86	CGTACGGTCGACGCTAGCTATTGATGTTAGCACGTGGAGCTCGGATCC	2.60	42.28
87	CGTACGGTCGACGCTAGCTCACATCGCAATCACGTGGAGCTCGGATCC	2.60	73.65

88	CGTACGGTCGACGCTAGCGTGGGAAACTAACACGTGGAGCTCGGATCC	2.60	19.09
89	CGTACGGTCGACGCTAGCCAGGCCTATCGGCACGTGGAGCTCGGATCC	2.60	36.82
90	CGTACGGTCGACGCTAGCCTGCTGCAAAGCCACGTGGAGCTCGGATCC	2.60	27.27
91	CGTACGGTCGACGCTAGCTCCCGATCTTAGCACGTGGAGCTCGGATCC	2.60	28.64
92	CGTACGGTCGACGCTAGCGGTGTTCTGATGCACGTGGAGCTCGGATCC	2.60	34.10
93	CGTACGGTCGACGCTAGCTATAGACACCGGCACGTGGAGCTCGGATCC	2.60	42.28
94	CGTACGGTCGACGCTAGCGTATCAGCCGCCCACGTGGAGCTCGGATCC	2.60	24.55
95	CGTACGGTCGACGCTAGCTGCACTTCCCTGCACGTGGAGCTCGGATCC	2.60	36.82
96	CGTACGGTCGACGCTAGCGTAAGTTGTACACACGTGGAGCTCGGATCC	2.60	31.36
97	CGTACGGTCGACGCTAGCAGCTGCGCGAGGCACGTGGAGCTCGGATCC	2.60	23.18
98	CGTACGGTCGACGCTAGCTGCCTCTCTCTCCACGTGGAGCTCGGATCC	2.60	84.54
99	CGTACGGTCGACGCTAGCAAGATATACAGTCACGTGGAGCTCGGATCC	2.60	15.00
100	CGTACGGTCGACGCTAGCGACTATGTACCACACGTGGAGCTCGGATCC	2.60	53.18

References

1. J. Zhang, K. Kobert, T. Flouri and A. Stamatakis, *Bioinformatics*, 2014, **30**, 614-620.
2. K. K. Alam, J. L. Chang and D. H. Burke, *Molecular Therapy. Nucleic Acids*, 2015, **4**, 230.

Mass Spectra

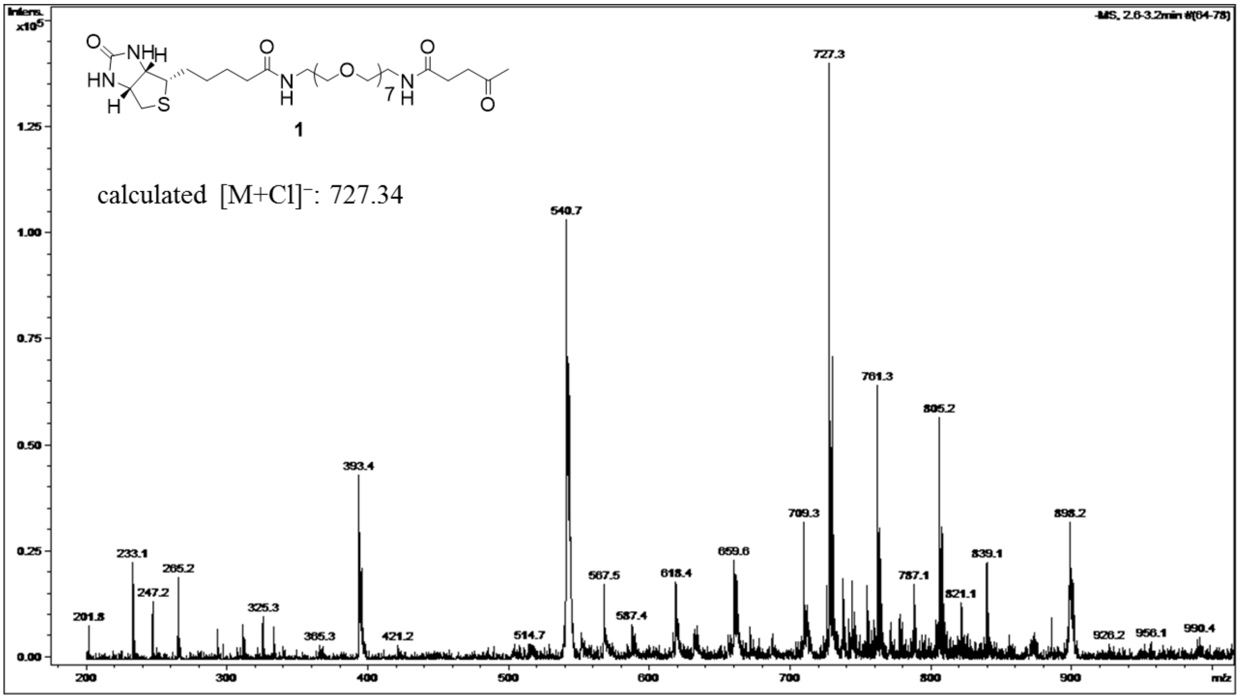

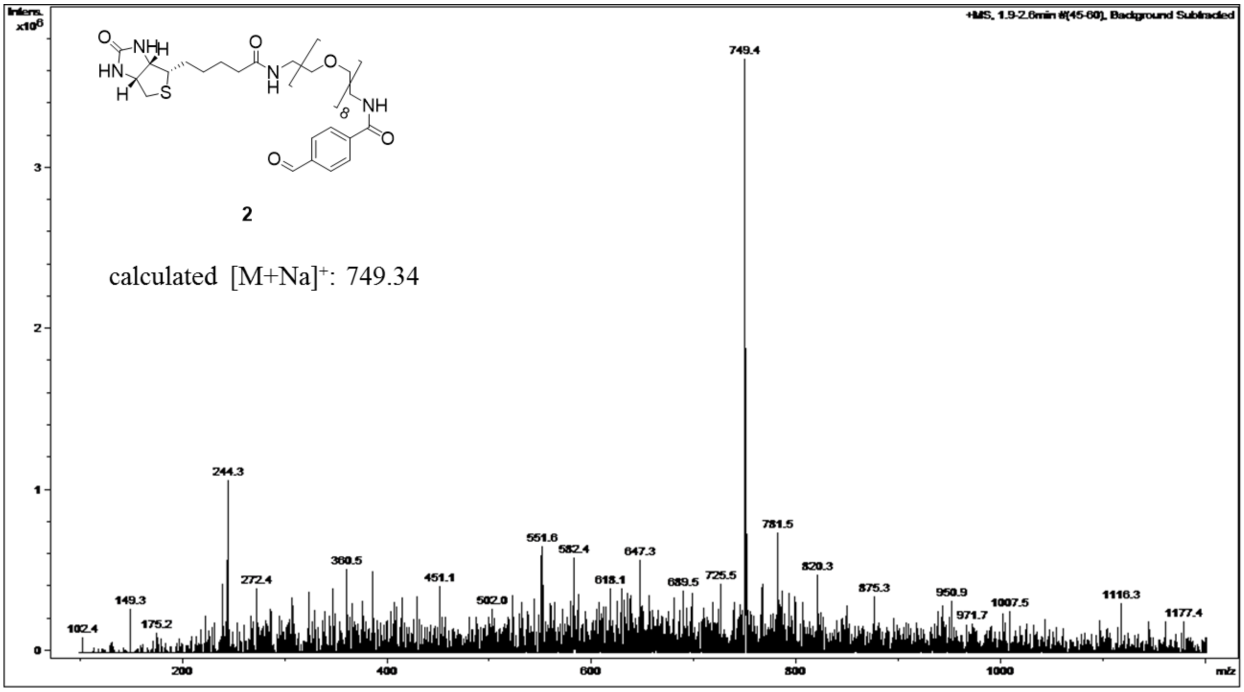

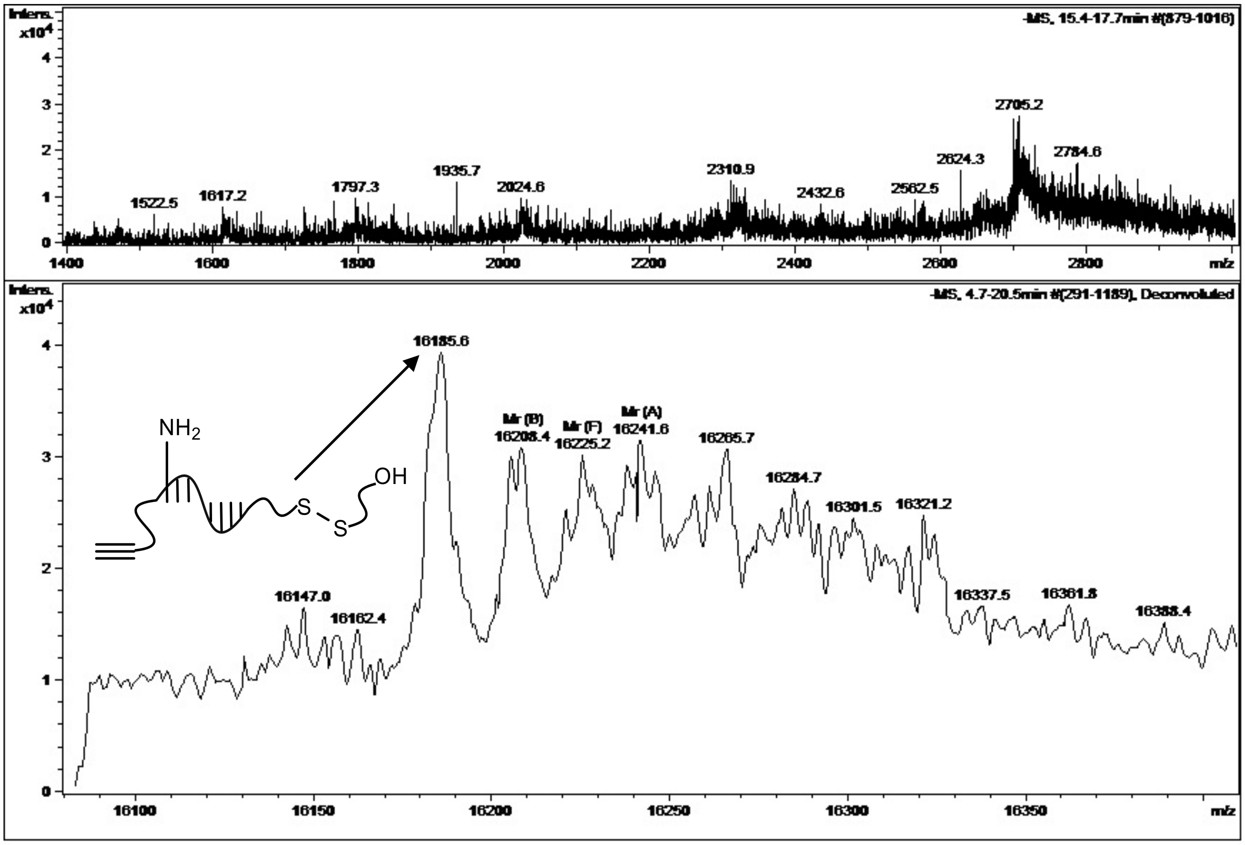

Intens.
x10^4
-MS, 15.4-17.7min #(879-1016)
1522.5
1617.2
1797.3
1935.7
2024.6
2310.9
2432.6
2562.5
2624.3
2705.2
2784.6
m/z
-MS, 4.7-20.5min #(291-1189), Deconvoluted
16185.6
Mr (B)
16208.4
Mr (F)
16225.2
Mr (A)
16241.6
16265.7
16284.7
16301.5
16321.2
16147.0
16162.4
16337.5
16361.8
16388.4
NH2
S
S
OH

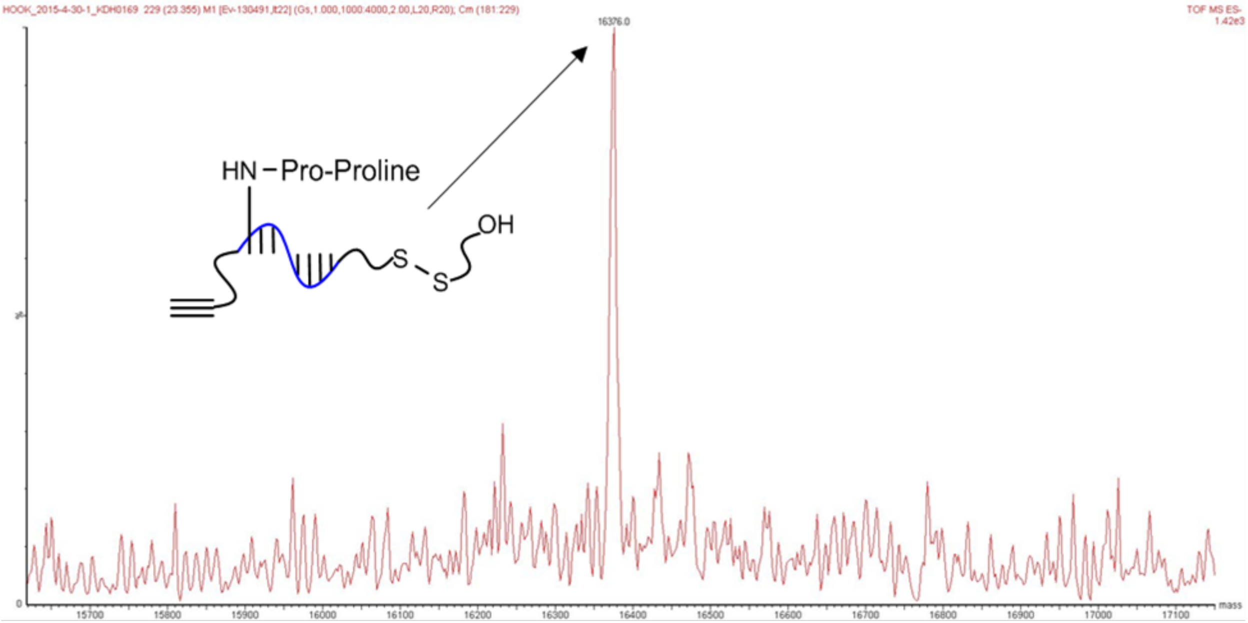

HOOK_2015-4-30-1_KDH0169 229 (23.355) M1 [Ev-130491,It22] (Gs,1.000,1000:4000,2.00,L20,R20); Cm (181:229)
TOF MS ES-
1.42e3
16376.0
HN−Pro-Proline
S
S
OH
mass

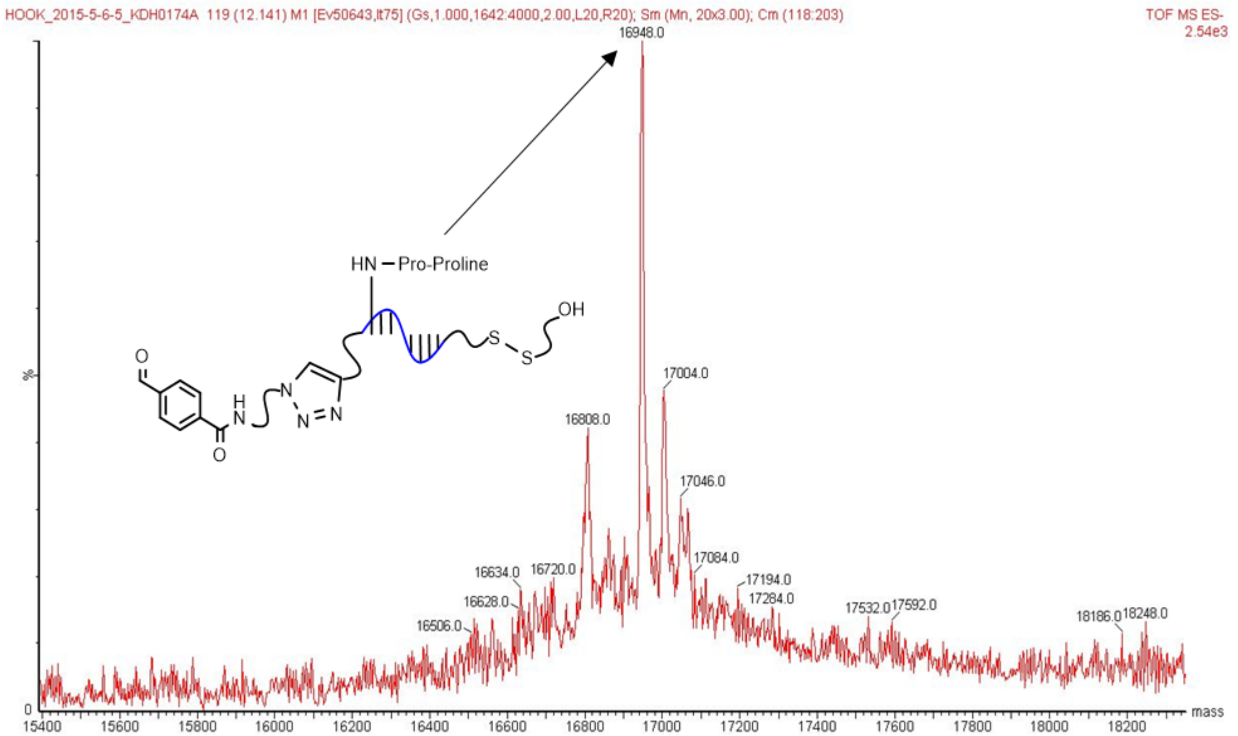
HOOK_2015-5-6-5_KDH0174A 119 (12.141) M1 [Ev50643,It75] (Gs,1.000,1642:4000,2.00,L20,R20); Sm (Mn, 20x3.00); Cm (118:203)
TOF MS ES-
2.54e3
16948.0
HN − Pro-Proline
OH
17004.0
16808.0
17046.0
17084.0
16634.0
16720.0
16628.0
16506.0
17194.0
17284.0
17532.0
17592.0
18186.0
18248.0
%
mass

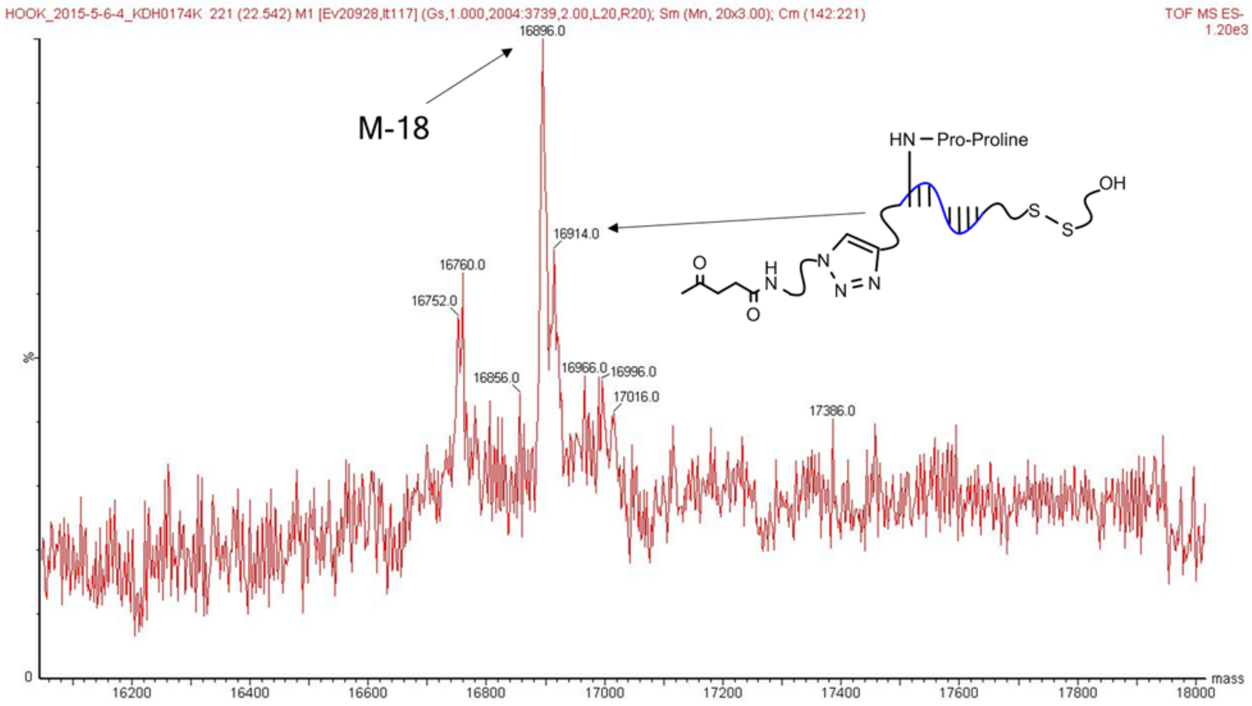
HOOK_2015-5-6-4_KDH0174K 221 (22.542) M1 [Ev20928,It117] (Gs,1.000,2004:3739,2.00,L20,R20); Sm (Mn, 20x3.00); Cm (142:221)
TOF MS ES-
1.20e3
16896.0
M-18
HN − Pro-Proline
OH
16914.0
16760.0
16752.0
16856.0
16966.0
16996.0
17016.0
17386.0
%
mass

PEGylated-oligonucleotides

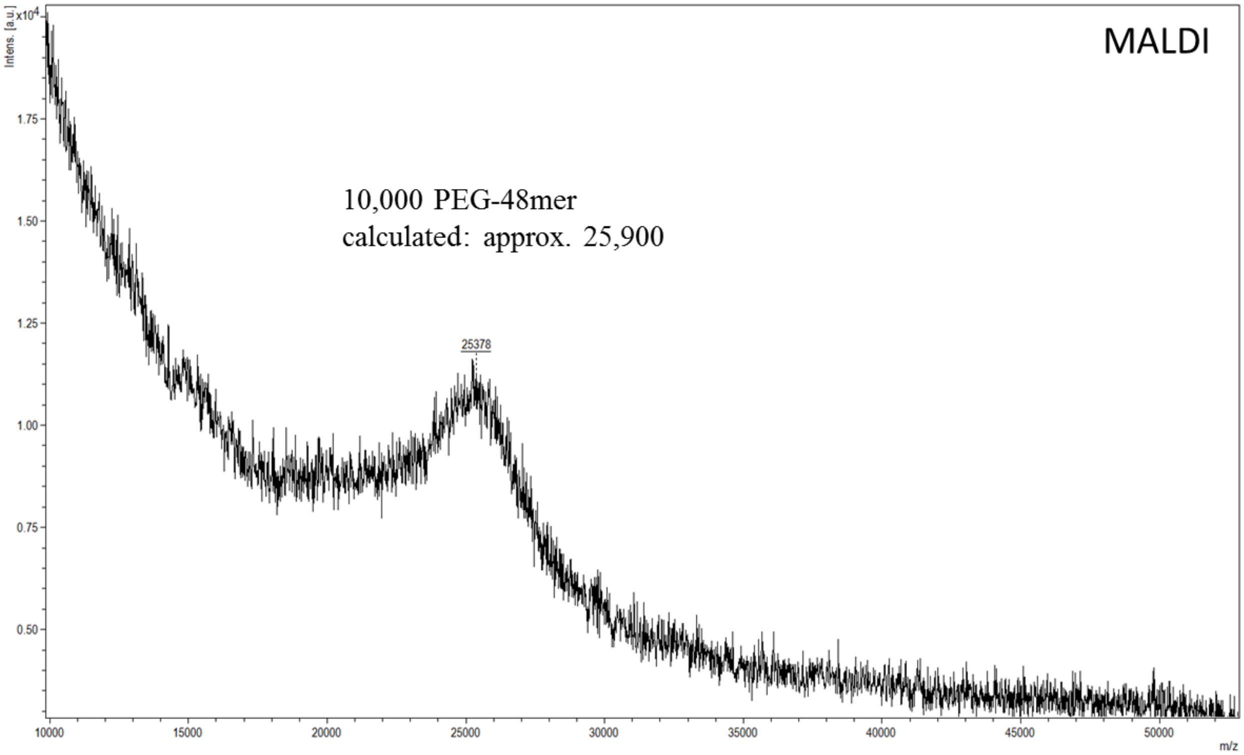

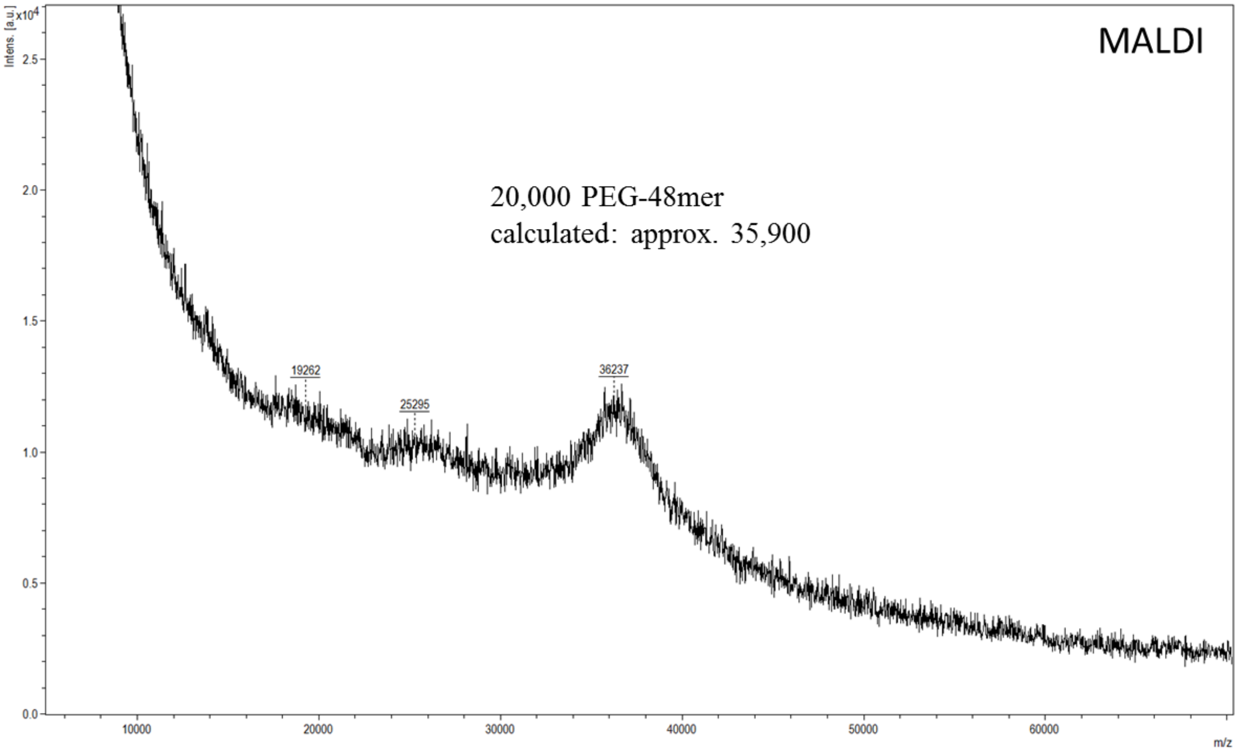

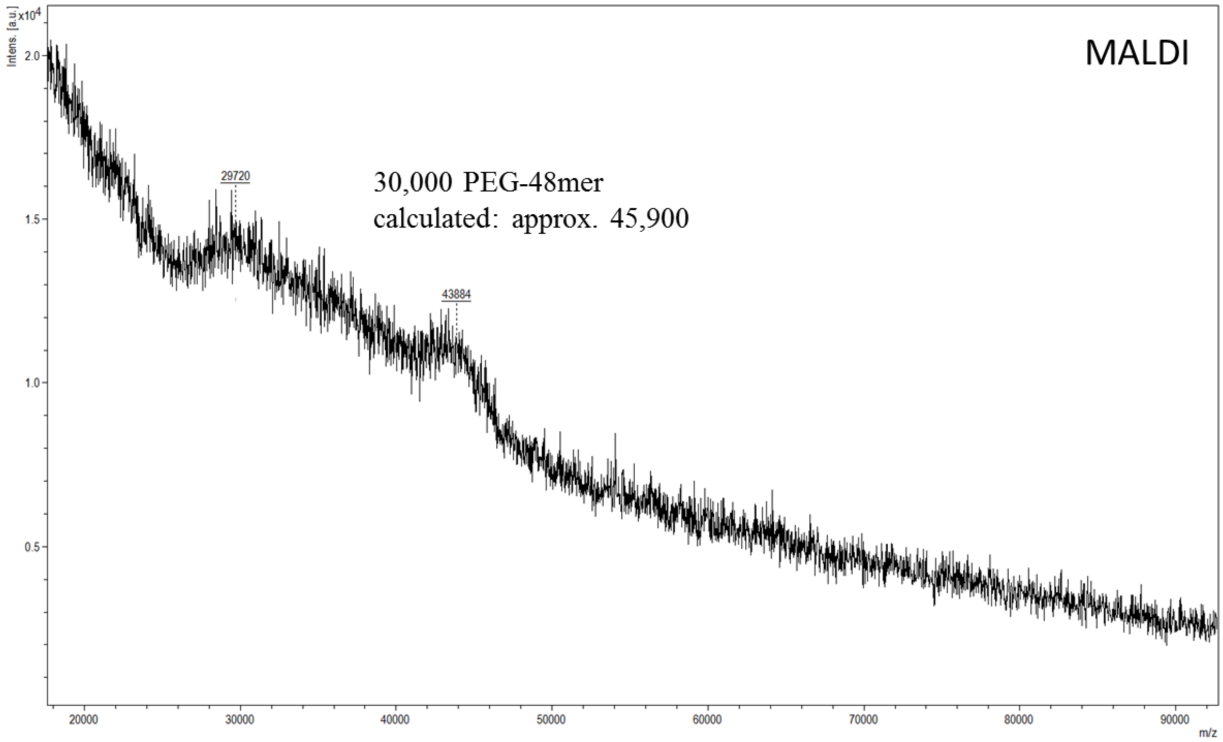
MALDI
30,000 PEG-48mer
calculated: approx. 45,900
29720
43884
Intens. [a.u.]
x10^4
m/z

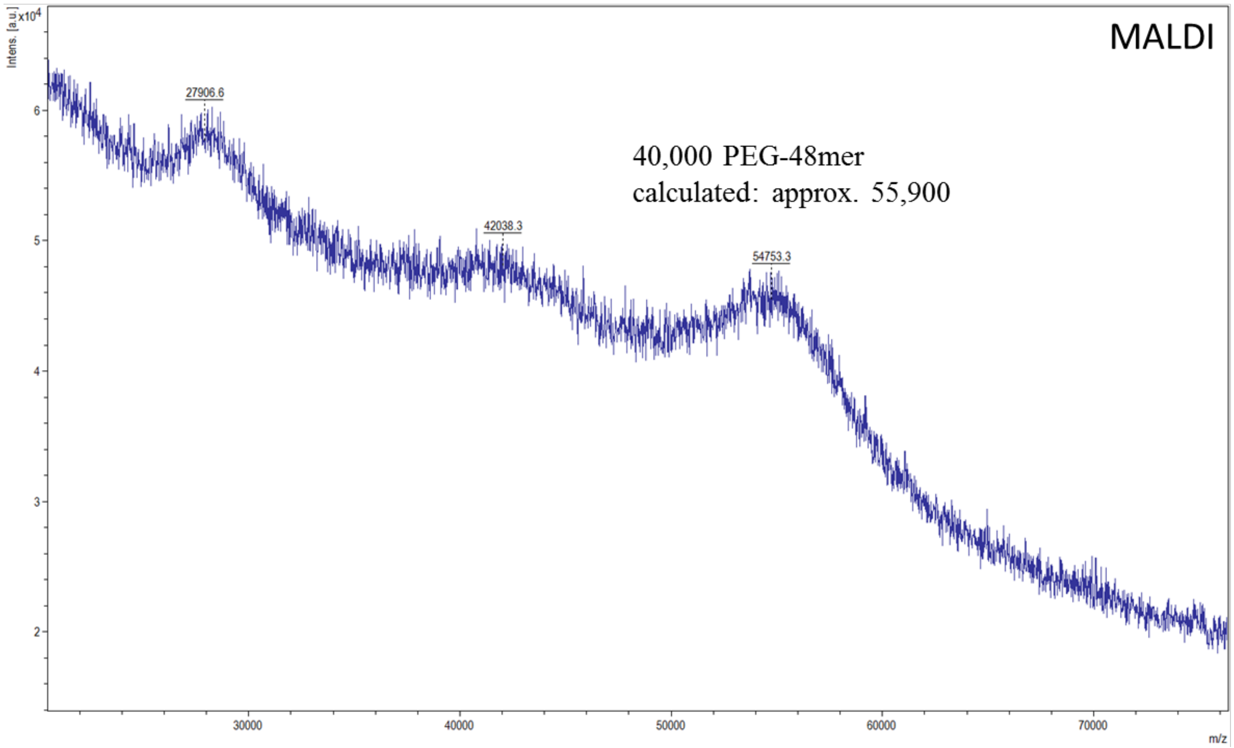
MALDI
40,000 PEG-48mer
calculated: approx. 55,900
27906.6
42038.3
54753.3
Intens. [a.u.]
x10^4
m/z
